# Multidimensional Impairment in Multiple Sclerosis: Physical Disability, Cognitive Dysfunction, Sleep Disturbance, Fatigue, Depression, and Their Impact on Quality of Life—A Possible Common Pathological Pathway

**DOI:** 10.3390/neurolint17110174

**Published:** 2025-10-22

**Authors:** Simona Petrescu, Maria-Melania Dumitru-Martoiu, Cristina Aura Panea

**Affiliations:** 1 Department of Neurology, Elias Emergency and University Hospital, 011461 Bucharest, Romania; simona.petrescu@umfcd.ro (S.P.); cristina.panea@umfcd.ro (C.A.P.); 2 Department of Clinical Neurosciences, University of Medicine and Farmacy, Carol Davila, 011461 Bucharest, Romania; 3 Nuffield Department of Clinical Neurosciences, University of Oxford, Oxford OX1 2JD, UK

**Keywords:** multiple sclerosis, quality of life, cognitive impairment, physical disability, fatigue, sleep quality, depression, patient-reported outcomes

## Abstract

Background: Multiple sclerosis (MS) is a chronic inflammatory disease of the central nervous system, which can lead to physical and cognitive disability, fatigue, depression, and sleep disturbance, all of which may impair quality of life (QoL). While the physical disability is widely known to influence the QoL, the relative contributions of cognitive impairment, fatigue, and sleep quality remain incompletely defined. Objectives: To evaluate the relationship between QoL, physical and cognitive disability, sleep quality, fatigue, and depression in people with MS (PwMS), and to explore phenotype-specific differences between relapsing and progressive forms. Methods: In this monocentric cross-sectional study, 112 PwMS underwent physical assessment (EDSS, MSFC), cognitive testing (SDMT, PASAT, MoCA, MMSE), and QoL evaluation (MSIS-29, EQ-5D, EQ-VAS, MSNQ). A subgroup of 29 patients also completed the Pittsburgh Sleep Quality Index (PSQI), Epworth Sleepiness Scale (ESS), Modified Fatigue Impact Scale (MFIS), and Beck Depression Inventory (BDI). Correlation and group analyses were performed. Results: Progressive MS patients showed greater physical disability (mean EDSS 5.8 vs. 2.6, *p* < 0.001), poorer cognitive performance, and lower QoL. Across the cohort, QoL strongly correlated with physical disability (EDSS) and cognitive performance (SDMT), with physical measures showing stronger associations. In relapsing MS, physical and cognitive impairment were linked to reduced QoL, whereas in progressive MS, physical disability predominated. In the sleep subgroup, poorer PSQI scores, longer sleep latency, and daytime sleepiness correlated with higher fatigue (MFIS), depressive symptoms (BDI), and reduced QoL (MSIS-29, EQ-5D). Conclusions: QoL in MS reflects the combined burden of physical disability, cognitive impairment, fatigue, depression, and poor sleep quality, with phenotype-specific patterns. While physical disability is the main QoL determinant in progressive MS, cognitive deficits with slowed processing speed play an important role in relapsing MS. Comprehensive, multidimensional assessment, including sleep and mood screening, may support individualized management strategies in MS.

## 1. Introduction

The concept of quality of life reflects the significance and evaluation of life by an individual. It can result from a personal and holistic assessment of one’s own life, and depends on the harmonious fulfillment of human needs, including health. In medicine, quality of life refers to an individual’s physical, psychological, and social well-being, as well as their ability to carry out daily activities [1].

Multiple sclerosis (MS) is a chronic inflammatory disease of the central nervous system and is the most common neurological disease in young adults, with onset most frequently occurring between the second and fourth decades [2]. MS leads to both physical and cognitive disability in this population [3], with a significant impact on individual life and society [4]. Axonal loss is an important component of disease progression, a phenomenon underlying disability in this condition. Therefore, evaluating the quality of life of this population is important, as it reflects the extent of the disease’s impact on an individual level [5].

The incidence and prevalence of MS have increased in recent years, even in regions previously considered low risk. This trend is associated with improvements in paraclinical evaluation techniques essential to the diagnostic process [6], as well as improved recognition capabilities and revision of diagnostic criteria [7].

To quantify neurological damage, clinical scales are used to measure the degree of clinical impairment and to assess therapeutic response (especially in clinical studies) [8]. The most widely used clinical scale is the EDSS (Expanded Disability Status Scale), developed by J. Kurtzke in 1983, after the clinical experience with the veterans of World War II, measuring disability across different functional scales [9].

However, the EDSS inadequately captures cognitive impairment, present in over 40% of MS patients, which prompted the development in 1999 of a more multidimensional scale. The MSFC (Multiple Sclerosis Functional Composite) [10]. The MSFC consists of three subscales assessing lower limb function (Timed 25-Foot Walk, T25FW), upper limb function (Nine-Hole Peg Test, 9HPT), and cognitive performance regarding attention, working memory and mathematical calculation domains (Paced Auditory Serial Addition Test, PASAT) [11].

Early clinical observations of frequent cognitive dysfunction in MS [12] led to the development of more specific tools to assess various cognitive domains. One such tool is the BICAMS (Brief International Cognitive Assessment for MS), composed of the Symbol Digit Modalities Test (SDMT), the California Verbal Learning Test-II (CVLT-II), and the Brief Visuospatial Memory Test-Revised (BVMT-R) [13].

Cognitive impairment in MS often begins with deficits in attention and information processing speed, and can progress to difficulties in working memory, planning, and decision-making [14]. These deficits tend to worsen as the disease progresses, correlating with disease duration, increased physical disability, and reduced cognitive reserve [15,16].

Objective tools like the Montreal Cognitive Assessment (MoCA) [17] have demonstrated utility in identifying cognitive dysfunction in MS across several studies [18]. MoCA is widely validated and used in multiple neurological conditions [19,20]. Another frequently used screening tool is the Mini-Mental State Examination (MMSE), which provides a general assessment of cognitive status by evaluating orientation, attention, memory, language, and visuospatial skills. Although MMSE is less sensitive than MoCA in detecting subtle cognitive deficits—particularly in early or mild cases of MS—it remains a commonly used instrument in clinical settings due to its simplicity and familiarity [21].

Subjective changes in cognition can also be captured by self-report tools like the Multiple Sclerosis Neuropsychological (MSNQ), which evaluates patient-perceived changes in cognitive status over the previous three months [22].

Quality of life in MS is typically assessed using self-reported questionnaires, aiming to capture the multiple deficits associated with the disease. Among the most commonly used are the Multiple Sclerosis Impact Scale (MSIS) and the Modified Fatigue Impact Scale (MFIS). MSIS-29 evaluates the patient’s perceived impact of MS on daily life over the past two weeks, with a total score between 29 and 145 [23]. MFIS assesses fatigue experienced in the past four weeks, covering physical, cognitive, and psychosocial dimensions, with a total score ranging from 0 to 84 [24].

In addition to MS-specific tools, the EuroQol five-dimension index score (EQ-5D), a general health-related quality of life questionnaire developed by the EuroQol group, is also used. It evaluates five health dimensions with different levels of impairment (3–5 levels): mobility, self-care, usual activities, pain/discomfort, and anxiety/depression [25]. Along with these questions, the patient gives himself a grade from 0 to 100, the limits being lower as the worst state of health and higher as the best state of health—EuroQol VAS (EQ VAS) scale [26,27].

Sleep, an essential component of daily well-being [28], is frequently disrupted in individuals with MS, particularly in association with fatigue, a commonly reported symptom. Recent studies support the presence of impaired sleep patterns in PwMS [29,30].

Sleep quality is defined by the individual’s subjective satisfaction with their sleep experience. It includes parameters such as sleep efficiency, latency, duration, and frequency of awakenings after sleep onset [31]. The Pittsburgh Sleep Quality Index (PSQI), a validated self-report instrument, is widely used to assess these aspects of sleep [32,33]. The daytime consequences of poor sleep are captured using the Epworth Sleepiness Scale (ESS), a self-report questionnaire that measures daytime somnolence. Its score ranges from 0 to 24, with scores above 13 indicating moderate, and scores above 16 indicating severe daytime sleepiness [34].

Depression is closely related to both sleep disturbance and fatigue in PwMS, each of these symptoms being capable of being influenced and amplified by the other. This triad often creates a complex that can significantly impact the quality of life. Among the available tools, the Beck Depression Inventory (BDI) is one of the most practical and widely used self-report instruments for assessing the severity of depressive symptoms in PwMS. It provides a quick, standardized, and sensitive measure of depression, facilitating its recognition and correlation with other symptoms such as fatigue and poor sleep quality [35,36].

Importantly, all these features—physical disability, cognitive dysfunction, fatigue, sleep disturbance, depression, and impaired quality of life—are ultimately linked to the underlying neuropathological hallmark of multiple sclerosis: axonal injury [37]. Axonal damage, resulting from inflammatory demyelination and neurodegenerative processes, represents the key driver of clinical symptoms and long-term neurological disability in MS. Therefore, assessing quality of life and cognitive function in MS not only reflects symptomatic burden but also indirectly captures the extent of axonal injury and disease progression [38,39].

## 2. Materials and Methods

### 2.1. Study Objectives

The primary objective of the study was to evaluate the self-reported quality of life in patients with multiple sclerosis (PwMS), specifically in relation to cognitive impairment as assessed by multiple neuropsychological tests. We aimed to determine whether differences in quality of life could be identified between cognitive and physical disability across different clinical phenotypes of the disease.

We conducted an exhaustive and objective evaluation of a representative cohort of PwMS. Physical disability was quantified using the Expanded Disability Status Scale (EDSS) and the physical subscales of the Multiple Sclerosis Functional Composite (MSFC), while cognitive impairment was assessed using standardized tests such as the Symbol Digit Modalities Test (SDMT), Paced Auditory Serial Addition Test (PASAT), Montreal Cognitive Assessment (MoCA), and Mini-Mental State Examination (MMSE).

A second objective of the study was to assess the impact of MS on sleep quality using the Pittsburgh Sleep Quality Index (PSQI) and daytime sleepiness using the Epworth Sleepiness Scale (ESS). Additionally, we investigated the correlations with sleep disturbance and other relevant clinical factors like fatigue and depression. Fatigue and its subcomponents were assessed using the Modified Fatigue Impact Scale (MFIS), while depressive symptoms were evaluated with the Beck Depression Inventory (BDI).

We conducted a quantitative and qualitative assessment of the sleep in PwMS by analyzing sleep parameters over the last month, including sleep and wake times, total sleep duration, sleep latency, and nighttime disturbances (e.g., pain or nocturia). These variables were evaluated alongside physical and cognitive disability, depression severity, daytime sleepiness, and fatigue level, all measured using validated and dedicated scales.

### 2.2. Ethical Considerations and Data Collections

All clinical evaluations were part of the patient’s annual follow-up. Data were obtained from the records of the National Program for Multiple Sclerosis. Neurological assessments and questionnaire completion were performed outside of any clinical relapse and in the absence of corticosteroid treatment, ensuring that results reflected a stable phase of the disease.

The study protocol was conducted in accordance with the Declaration of Helsinki. Patient consent was waived because only anonymized data were analyzed. The study was monocentric, performed at Elias University Emergency Hospital.

### 2.3. Methods

**Study population**

**Figure 1:**
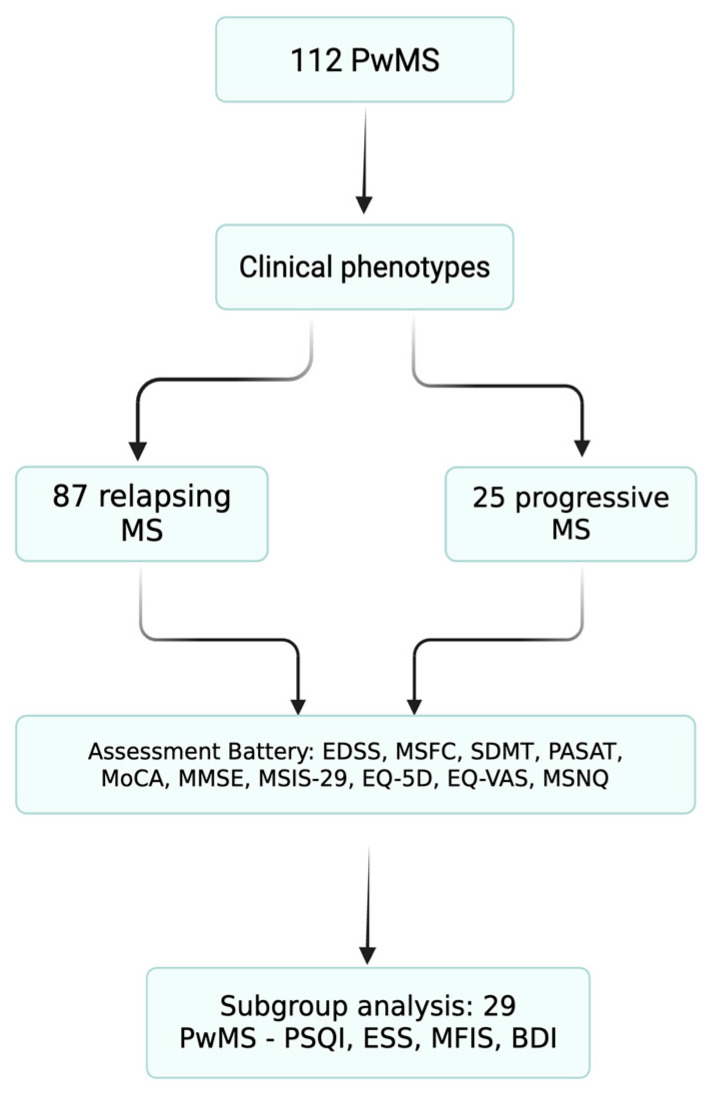
**Patient flow diagram.** Flow diagram of patient inclusion and subgroup allocation. A total of 112 people with multiple sclerosis (PwMS) were included from the Elias University Emergency Hospital database. Patients underwent standardized assessments of physical disability (EDSS and MSFC), cognition (SDMT, PASAT, MoCA, and MMSE), and quality of life (MSIS-29, EQ-5D, EQ-VAS, and MSNQ). A consecutive subgroup of 29 relapsing patients additionally completed sleep and mood questionnaires (PSQI, ESS, MFIS, and BDI).

To address the multiple aspects of quality of life in PwMS, a disease with a highly heterogeneous clinical presentation, we analyzed clinical and demographic data from the database of the Multiple Sclerosis Center of Elias University Emergency Hospital, a monocentric cohort. The evaluations were conducted in two phases, as shown in Figure 1.

At the initial assessment, patients underwent a thorough clinical evaluation, including measurement of physical disability, using the EDSS score. Motor function was evaluated through physical subtests of MSFC, specifically the T25FW for lower limb functions and the 9HPT for the upper limb function (dominant and non-dominant hand).

Cognitive assessment included both general and MS-specific tools. General cognitive function was screened using the MoCA and the MMSE scores. To specifically capture the cognitive domains frequently affected in MS, we also administered the PASAT and the SDMT scores.

To evaluate quality of life, we used different self-report questionnaires. General health-related quality of life was assessed using the EQ-5D index score and the EQ VAS scale. The disease-specific impact on quality of life was measured with the MSIS-29 scale. To identify self-perceived cognitive difficulties, patients completed the MSNQ questionnaire.

Patients were classified according to clinical phenotype into two main categories: relapsing forms, including clinically isolated syndrome (CIS) and relapsing-remitting multiple sclerosis (RRMS), and progressive forms, including secondary progressive multiple sclerosis (SPMS) and primary progressive multiple sclerosis (PPMS).

In total, 112 patients were included in the study: 77 females and 35 males. Of these, 87 were classified as having relapsing forms and 25 as having progressive forms in MS.

**Sleep Sub-study**

To assess sleep quality, a subgroup of 29 MS completed the PSQI index and ESS scale. Additional assessments included the MFIS scale and Beck Depression Inventory (BDI), in order to better define the link between sleep quality, fatigue, and depression in MS.

### 2.4. Data Analysis

Data acquisition, centralization, and statistical analysis were performed with Statistical Package for the Social Sciences (version 26.0, SPSS Inc., Chicago, IL, USA), while the manuscript was drafted using Microsoft Word 2020.

The following analytical and descriptive statistical methods were applied: Pearson correlation coefficient—to evaluate direct or inverse proportional relationship between two sets of quantitative variables, Levene two-sample *t*-test—to compare means between two groups, and ANOVA parametric test—to analyze the relationship between the dependent variable and multiple categories of independent variables.

## 3. Results

### 3.1. Demographic and Clinical Characteristics

The study included 112 patients diagnosed with MS: 77 women (68.7%) and 35 men (31.3%), with a mean age of 38.1 years (range 16–60). A total of 87 patients (77.7%) were diagnosed with relapsing MS (CIS or RRMS), while 25 patients (22.3%) had progressive MS (SPMS and PPMS) (Table 1).

The mean EDSS score across the cohort was 3.34 (range 0–7.0), with women showing a slightly higher disability (mean EDSS 3.4) compared to men (mean EDSS 3.3). Relapsing forms had significantly lower disability (mean EDSS 2.6), compared to progressive forms (mean EDSS 5.8).

### 3.2. Physical Disability and Cognitive Function

Significant negative correlations were observed between EDSS and cognitive test scores across the whole cohort (*p = 0.0001*), indicating that increased physical disability is associated with poorer cognitive performance. The strongest associations were observed between EDSS-SDMT (r= −0.587) and EDSS-PASAT (r= −0.466) (Table 2).

### 3.3. QoL and Self-Report Measurements

MSIS-29 correlated positively with MSNQ (r = 0.622) and EQ-5D (r= 0.825), and negatively with EQ VAS (r= −0.774). Higher perceived disease burden was associated with a lower self-rated health (Table 3).

### 3.4. QoL, Physical Disability, and Cognitive Function

Both physical and cognitive impairments correlated with QoL outcomes (Table 4). Physical disability measures (EDSS, T25FW, 9HPT) showed stronger correlations overall. Cognitive tests (SMDT, PASAT) also correlated significantly, particularly with MSIS-29 and EQ-5D.

### 3.5. Relapsing vs. Progressive Subgroups—QoL, Physical Disability, and Cognitive Function

Patients with progressive MS (2) scored consistently lower on all cognitive tests (SDMT, PASAT, MoCA, and MMSE) and reported greater QoL impairment (MSIS-29, EQ-5D, EQ VAS, MSNQ) compared with relapsing MS (1) (Table 5).

In relapsing MS, disability score (EDSS) correlated with cognitive test measures (SDMT, PASAT, MMSE). The MoCA showed a trend (p = 0.053), not reaching statistical significance (Table 6). QoL correlated with both cognitive and physical disability measurements (Table 7).

In progressive MS, there were no statistically significant correlations between disability score and cognitive test, possibly due to the smaller sample size (Table 6). QoL correlated mainly with EDSS, while associations with cognitive tests did not reach statistical significance (Table 7).

### 3.6. Subgroup Analysis of Sleep Quality (29 Patients)

A subgroup of 29 patients (32.48%, 21F:8M), with relapsing MS, completed additional questionnaires for sleep quality (PSQI), daytime sleepiness (ESS), fatigue (MFIS), and depression (BDI).

In this subgroup, MSIS-29 correlated with MFIS total (*p = 0.0002*), BDI (*p < 0.001*), sleep scores (ESS *p < 0.001*, PSQI *p = 0.005*), while EQ-5D correlated only with MFIS, BDI and PSQI, and EQ VAS correlated with MFIS and BDI (Table 8).

ESS correlated positively with PSQI (*p = 0.0001*) and MFIS total (*p = 0.0001*). MFIS correlated with PSQI (*p < 0.0001*).

BDI correlated with ESS (*p = 0.003*) and MFIS total (*p < 0.0001*), sleep latency (*p = 0.002*), and bedtimes (*p < 0.0001*).

### 3.7. ANOVA Analysis of Sleep Disturbance

Further ANOVA analysis was performed on PSQI questions related to sleep latency and sleep maintenance (no event, under 1 event, 1–2 events, and 3 or more events per week during the last month). Patients who had difficulty falling asleep ≥3 times per week reported significantly higher scores on MSIS-29 (*p = 0.0005*), BDI (*p = 0.0006*), ESS (*p = 0.003*), and MFIS (physical *p = 0.003* and cognitive *p = 0.0002*) (Table 9a).

Nocturnal awakenings also correlated with increased depression (BDI, *p = 0.0007*) and fatigue (MFIS total, *p = 0.0001*) (Table 9b). In most cases, these awakenings were attributed to nighttime disturbances such as pain or nocturia.

## 4. Discussion

The demographic and clinical characteristics of our study group are consistent with known epidemiological patterns in MS [40]. Women represented the majority of cases, with a female-to-male ratio of 2.2:1 and a mean age of 38.1 years. This aligns with global data showing a higher incidence in women, especially in relapsing forms of the disease [41]. Notably, a higher proportion of men was observed in the progressive MS group (40%) compared to the relapsing group (28.7%), reinforcing prior observations that male sex may be associated with a more aggressive disease course and worse prognosis [42]. The mean EDSS score of 3.3 reflects a population with moderate physical disability. Subgroup analysis revealed a significantly higher disability burden in patients with progressive MS (mean EDSS = 5.8) compared to those with relapsing MS (mean EDSS = 2.6), consistent with expected disease trajectories and previously published data [43,44,45,46].

Our findings demonstrate that QoL in MS patients are influenced by both physical and cognitive disability, regardless of the self-report tools used—whether general measurements like EQ-5D (including the VAS score) or disease-specific tools such as MSIS-29 and MSNQ [47]. This is consistent with prior studies, yet our analysis distinguishes itself through the comprehensive and objective evaluation of both physical (EDSS, MSFC) and cognitive (SDMT, PASAT, MoCA, and MMSE) function, and their correlation with patient perceived QoL.

In contrast to other studies focusing on one dimension, our approach offers a multifaceted perspective, incorporating both clinician and patient-reported outcomes [48,49,50].

It confirms the multidimensional burden of MS, integrating physical disability, cognitive dysfunction, fatigue, depression, and sleep disturbance, all of which contribute to reduced QoL.

In relapsing MS, QoL is significantly impacted by both physical and cognitive deficits [51], particularly by early cognitive changes such as reduced information processing speed and impaired working memory [52]. However, in progressive MS, QoL was more strongly related to physical disability, though lack of significant cognitive associations may reflect limited statistical power with a possible risk of type II error in subgroup analysis (n = 25), rather than the absence of the effect [53]. These findings support the clinical relevance of distinguishing between MS phenotypes in patient management and rehabilitation strategies [54].

Fatigue, poor sleep quality, and depression formed a strongly interrelated cluster in our study. This triad has been reported in prior MS research and may share underlying mechanisms such as neuroinflammation, hypothalamic dysfunction, circadian disruption, and impaired neurotransmitter regulation. The high expression of these symptoms may contribute disproportionately to reduced QoL, independent of physical disability. Clinically, this emphasizes the importance of integrated screening and management of mood, fatigue, and sleep disturbance in PwMS [55].

In our study, sleep quality (assessed by using the PSQI and ESS) was found to be impaired, consistent with recent evidence [56]. Although limited by the small sample size, our analyses suggest a multifactorial origin of poor sleep quality, involving pain, nocturia, fatigue, and mood disturbances. In addition, prior research indicates that lesion location and axonal damage in sleep-regulating brain regions such as the hypothalamus and brainstem may contribute to this clinical picture [57,58]. Beyond these clinical and structural contributors, growing evidence suggests that sleep impairment itself may exacerbate neurodegeneration and accelerate cognitive decline, through mechanisms like increased neuroinflammation and altered synaptic plasticity. Sleep has thus emerged as a potentially modifiable factor influencing MS outcomes [59,60]. Our findings, limited to a small subgroup, align with this emerging literature and reinforce the need to integrate sleep evaluation and management into comprehensive MS care.

The MSIS-29 proved especially valuable, as it incorporates dimensions often omitted by traditional disability scales, such as fatigue, sleep impairment, and depressive symptoms [61]. This supports its utility as a comprehensive QoL measure in clinical and research settings [62].

In line with previous literature, depressive symptoms were more prevalent in patients reporting daytime sleepiness and higher levels of physical and cognitive fatigue [63]. Depression not only affects QoL directly but also serves as a significant mediator of poor sleep quality, associated with sleep fragmentation and prolonged sleep latency [50,64]. Our findings emphasize the need for routine assessment and management of mood and sleep disorders in MS care.

The study’s strengths include its broad, multidimensional design, integrating comprehensive assessment using objective clinical tests (EDSS, MSFC, and cognitive batteries) with validated self-report questionnaires (EQ-5D, MSIS-29, MFIS, PSQI, and MSNQ) [65]. This comprehensive approach enabled integration of physical, cognitive, sleep and mood domains and allowed for phenotype-specific analysis, distinguishing the relative contributions of cognitive and physical disability to QoL in relapsing vs. progressive MS. The use of multiple cognitive instruments, including MoCA and MMSE, strengthened the reliability of the cognitive assessment.

However, several limitations must be acknowledged. First, the cross-sectional design of the study without longitudinal follow-up may limit the ability to assess causal relationships or limit conclusions regarding the long-term progression of symptoms. Second, the monocentric design may limit the possibility of generalizing to broader or more diverse populations with different care settings, ethnic backgrounds, or health systems. Third, the sleep-focused analysis was restricted to a small subgroup of 29 patients, which reduces the statistical power for analyzing sleep-related correlations and raises the risk of type II error. Moreover, data on sleep, fatigue, and depression were not available for all patients, limiting the power of subgroup analyses. Fourth, no correction for multiple testing was applied, increasing the likelihood of false-positive associations. Fifth, uneven sample sizes across phenotypes (25 progressive vs. 87 relapsing) reduce statistical power in subgroup comparisons. Sixth, several important covariates, including disease duration, comorbidities, and treatment type, were not fully available, which may have influenced associations. Finally, the absence of neuroimaging data prevented direct correlation between lesion burden, axonal injury, and clinical outcomes.

**Figure 2:**
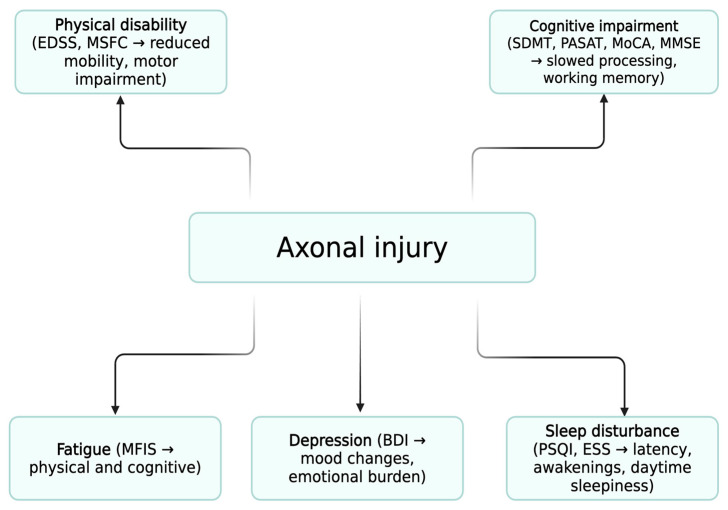
**Conceptual Model of Multidimensional Impairment in MS**. Schematic representation of the proposed “common pathological pathway.” Axonal injury is shown as the central pathological mechanism linked to physical disability, cognitive impairment, fatigue, sleep disturbance, and depression. These domains converge to determine quality of life (QoL). Fatigue, sleep disturbance, and depression form an interrelated triad with reciprocal interactions, amplifying their contribution to reduced QoL.

Nonetheless, the observed associations between cognitive and physical disability and reduced QoL, particularly in relation to fatigue, mood and sleep disturbance, are consistent with the concept that both inflammatory and neurodegenerative mechanisms, particularly axonal injury, play a central role in the progression of MS-related disability. Axonal damage underlies both physical disability and cognitive decline, reinforcing the need for neuroprotective and multidimensional strategies in MS management (Figure 2) [62].

## 5. Conclusions

This study highlights the complex and interdependent relationships between physical disability, cognitive impairment, fatigue, depression, and sleep quality in multiple sclerosis. Quality of life in MS is not determined by a single factor but reflects the cumulative burden of multiple domains.

While physical disability remains the primary determinant in progressive MS, cognitive deficits, particularly slowed information processing speed, play a more significant role in relapsing forms. Importantly, our exploratory subgroup findings suggest that poor sleep quality, fatigue, and depression form a tightly linked triad that further exacerbates the patient-reported burden.

At the biological level, these multidimensional impairments can be understood as converging consequences of axonal injury, the key pathological hallmark of MS. This unifying mechanism underscores the need for neuroprotective strategies while also justifying multidimensional clinical assessment.

Given the multifactorial burden of MS, we recommend the routine use of comprehensive assessment tools, along with EDSS for physical disability, the use of patient-reported outcomes for QoL, such as the MSIS-29 and EQ-5D, SDMT or MoCA for cognitive screening, PSQI and ESS for sleep assessment, MFIS for fatigue evaluation, and BDI for depression assessment.

Such a streamlined protocol could be administered annually, guiding individualized, multidisciplinary interventions targeting not only motor but also non-motor determinants of QoL.

## Figures and Tables

**Table 1:** Demographic and clinical data.

	N	F (N, p%)	M (N, p%)	EDSS (Mean, Min–Max)
**Total**	112	77 (68.7%)	35 (31.3%)	3.34 (0–7.0)
**Mean age** **(min–max)**	38.1 (16–60)	40.1 (18–60)	33.6 (16–55)	/
**Relapsing forms**	87 (77.7%)	62 (71.3%)	25 (28.7%)	2.6 (0–6.0)
**Progressive forms**	25 (22.3%)	15 (60%)	10 (40%)	5.8 (3.0–7.0)

Abbreviations: N—number, p%—percent, EDSS—Expanded Disability Status Scale.

**Table 2:** Physical disability and cognitive function measurements.

		SDMT	PASAT	MoCA	MMSE
**EDSS**	*p*	0.0001	0.0001	0.0001	0.0001
	r	−0.587	−0.466	−0.390	−0.400

Abbreviations: SDMT—Symbol Digit Modalities Test, PASAT—Paced Auditory Serial Addition Test, MoCA—Montreal Cognitive Assessment, MMSE—Mini-Mental State Examination.

**Table 3:** QoL and self-report measurements.

		MSNQ	EQ-5D	EQ VAS
**MSIS** **-29**	*p*	0.0001	0.0001	0.0001
	r	0.622	0.825	−0.774

Abbreviations: MSIS-29—Multiple Sclerosis Impact Scale 29, MSNQ—Multiple Sclerosis Neuropsychological, EQ-5D—EuroQol five-dimension index score, EQ VAS—EuroQol Visual Analogue Scale.

**Table 4:** QoL, physical disability and cognitive function measurements.

		SDMT	PASAT	MoCA	MMSE	EDSS	9HPT DH	9HPT NDH	T25FW
**MSIS–29**	*p*	0.0001	0.0001	0.0001	0.0001	0.0001	0.0001	0.0001	0.0001
	r	−0.544	−0.520	−0.314	−0.491	0.686	0.579	0.610	0.547
**EQ**-**5D**	*p*	0.0001	0.0001	0.0001	0.0001	0.0001	0.0001	0.0001	0.0001
	r	−0.574	−0.524	−0.375	−0.511	0.683	0.593	0.589	0.526
**EQ VAS**	*p*	0.0001	0.0001	0.0001	0.0001	0.0001	0.0001	0.0001	0.0001
	r	0.524	0.471	0.336	0.402	−0.683	−0.518	−0.542	−0.515

Abbreviations: SDMT—Symbol Digit Modalities Test, PASAT—Paced Auditory Serial Addition Test, MoCA—Montreal Cognitive Assessment, MMSE—Mini-Mental State Examination, MSIS 29—Multiple Sclerosis Impact Scale 29, EQ-5D—EuroQol five-dimension index score, EQ VAS—EuroQol Visual Analogue Scale, EDSS—Expanded Disability Status Scale, 9HPT—9-Hole Peg Test, DH—Dominant Hand, NDH—Non-Dominant Hand, T25FW—25-Foot Walk Test.

**Table 5:** Cognitive Function and QoL between groups (Levene test).

		1	2	*p* Value
**Total N**	112	87	25	/
**SDMT m**	38.25	41.61	25.52	<0.001
**PASAT m**	43.32	46.05	33.84	<0.001
**MoCA m**	23.62	24.67	19.96	<0.001
**MMSE m**	27.96	28.44	26.28	0.004
**MSIS-29 m**	59.67	51.91	86.24	<0.001
**MSNQ m**	15.89	13.92	22.68	0.009
**EQ-5D m**	2.32	1.69	4.48	<0.001
**EQ VAS m**	71.04	76.21	53.24	<0.001

Abbreviations: SDMT—Symbol Digit Modalities Test, PASAT—Paced Auditory Serial Addition Test, MoCA—Montreal Cognitive Assessment, MMSE—Mini-Mental State Examination, MSIS 29—Multiple Sclerosis Impact Scale 29, MSNQ—Multiple Sclerosis Neuropsychological, EQ-5D—EuroQol five-dimension index score, EQ VAS—EuroQol Visual Analogue Scale.

**Table 6:** Physical disability and cognitive function between groups.

			SDMT	PASAT	MMSE	MoCA
**1**	**EDSS**	*p*	0.0001	0.001	0.006	0.053
		r	−0.488	−0.361	−0.294	−0.208
**2**	**EDSS**	*p*	0.491	0.012	0.035	0.061
		r	−0.151	−0.492	−0.422	−0.380

Abbreviations: SDMT—Symbol Digit Modalities Test, PASAT—Paced Auditory Serial Addition Test, MoCA—Montreal Cognitive Assessment, MMSE—Mini-Mental State Examination, EDSS—Expanded Disability Status Scale.

**Table 7:** QoL, physical disability and cognitive function between groups.

			SDMT	PASAT	MoCA	MMSE	EDSS	9HPT d	9HPT n	T25FW
	**MSIS-29**	*p*	0.0001	0.0001	0.173	0.0001	0.0001	0.0001	0.0001	0.0001
		r	−0.496	−0.464	−0.148	−0.485	−0.583	0.423	0.500	0.492
**1**	**EQ-5D**	*p*	0.0001	0.0001	0.056	0.0001	0.0001	0.0001	0.0001	0.0001
		r	−0.476	−0.427	−0.207	−0.455	0.528	0.443	0.431	0.462
	**EQ VAS**	*p*	0.0001	0.0001	0.086	0.003	0.0001	0.0001	0.0001	0.0001
		r	0.488	0.380	0.186	0.312	−0.602	−0.408	−0.419	−0.499
	**MSIS–29**	*p*	0.587	0.194	0.449	0.258	0.003	0.202	0.087	0.108
		r	−0.122	−0.269	−0.159	−0.235	0.576	0.264	0.349	0.391
**2**	**EQ-5D**	*p*	0.186	0.086	0.087	0.050	0.002	0.089	0.029	0.519
		r	−0.286	−0.350	−0.349	−0.397	0.595	0.347	0.438	0.163
	**EQ VAS**	*p*	0.951	0.221	0.307	0.209	0.092	0.969	0.317	0.763
		r	−0.014	0.254	0.213	0.260	−0.344	0.008	−0.208	−0.076

Abbreviations: SDMT—Symbol Digit Modalities Test, PASAT—Paced Auditory Serial Addition Test, MoCA—Montreal Cognitive Assessment, MMSE—Mini-Mental State Examination, MSIS-29—Multiple Sclerosis Impact Scale 29, EQ-5D—EuroQol five-dimension index score, EQ VAS—EuroQol Visual Analogue Scale, EDSS—Expanded Disability Status Scale, 9HPT—Nine-Hole Peg Test, DH—Dominant Hand, NDH—Non-Dominant Hand, T25FW—25-Foot Walk Test.

**Table 8:** QoL, fatigue, depression, daytime sleepiness, and sleep quality.

		MFIS p	MFIS c	MFIS t	BDI	ESS	PSQI
**MSIS-29**	*p*	0.001	0.0003	0.0002	<0.001	<0.001	0.005
**EQ-5D**	*p*	0.045	0.013	0.014	0.005	0.652	0.147
**EQ VAS**	*p*	0.002	<0.001	<0.001	0.029	0.115	0.049

Abbreviations: MSIS-29—Multiple Sclerosis Impact Scale 29, EQ-5D—EuroQol five-dimension index score, EQ VAS—EuroQol Visual Analogue Scale, EDSS—Expanded Disability Status Scale, MFIS—Modified Fatigue Impact Scale, p—physical, c—cognitive, t—total, BDI—Beck Depression Inventory, ESS—Epworth Sleepiness Scale, PSQI—Pittsburgh Sleep Quality Index.

**Table 9:** PSQI: sleep latency/awakenings during night, QoL, depression and fatigue—ANOVA analysis.

**a—Prolonged Sleep Latency—First 30 min**						
**Group**	**Nr**	**MSIS-29**	**BDI**	**ESS**	**MFIS p**	**MFIS c**
**NO**	13	39.9	5.3	5.3	16.6	12.6
**<1/Week**	11	47.5	4.8	5.2	9.8	7.6
**1-2/Week**	1	16	20	12	39	29
**≥3/Week**	4	77	22	13.25	36.5	30.75
***p***		0.000572	0.000604	0.003256	0.000364	0.000212
**b—Awakenings During Night**						
**Group**	**Nr**	**BDI**	**MFIS t**			
**NO**	4	2.5	14			
**<1/Week**	12	2.3	16.4			
**1,2/Week**	4	16.26	47			
**≥3/Week**	9	14.1	51.3			
***p***		0.000734	0.000146			

Abbreviations: MSIS-29—Multiple Sclerosis Impact Scale 29, MFIS—Modified Fatigue Impact Scale, p—physical, c—cognitive, BDI—Beck Depression Inventory, ESS—Epworth Sleepiness Scale, PSQI—Pittsburgh Sleep Quality Index, t—total.

## Data Availability

The data used to support the findings of this study are included within the article.
